# Expectancy and surprise predict neural and behavioral measures of attention to threatening stimuli

**DOI:** 10.1016/j.neuroimage.2011.09.007

**Published:** 2012-01-16

**Authors:** Michael Browning, Catherine J. Harmer

**Affiliations:** Department of Psychiatry, Oxford University, Oxford, UK

**Keywords:** Attention, Emotion, Learning, Anxiety, fMRI

## Abstract

Attention is preferentially deployed toward those stimuli which are threatening and those which are surprising. The current paper examines the intersection of these phenomena; how do expectations about the threatening nature of stimuli influence the deployment of attention? The predictions tested were that individuals would direct attention toward stimuli which were expected to be threatening (regardless of whether they were or not) and toward stimuli which were surprising. As anxiety has been associated with deficient control of attention to threat, it was additionally predicted that high levels of trait anxiety would be associated with deficits in the use of threat-expectation to guide attention. During fMRI scanning, 29 healthy volunteers completed a simple task in which threat-expectation was manipulated by altering the frequency with which fearful or neutral faces were presented. Individual estimates of threat-expectation and surprise were created using a Bayesian computational model. The degree to which the model derived estimates of threat-expectation and surprise were able to explain both a behavioral measure of attention to the faces and activity in the visual cortex and anterior attentional control areas was then tested.

As predicted, increased threat-expectation and surprise were associated with increases in both the behavioral and neuroimaging measures of attention to the faces. Additionally, regions of the orbitofrontal cortex and left amygdala were found to covary with threat-expectation whereas anterior cingulate and lateral prefrontal cortices covaried with surprise. Individuals with higher levels of trait anxiety were less able to modify neuroimaging measures of attention in response to threat-expectation. These results suggest that continuously calculated estimates of the probability of threat may plausibly be used to influence the deployment of visual attention and that use of this information is perturbed in anxious individuals.

## Highlights

► Attention is directed toward stimuli which are expected to be threatening. ► Attention is also captured by surprising stimuli. ► The OFC and amygdala track threat-expectation. ► The ACC tracks the surprise associated with a stimulus. ► Anxious participants are less able to use threat-expectation to control attention.

## Introduction

Emotionally salient stimuli, particularly those which are threatening, attract attention (Vuilleumier, 2005). However, the deployment of attention is also influenced by expectations about the environment; infrequent or surprising stimuli capture attention to a greater extent than those which are expected (Itti and Baldi, 2009; Logan and Zbrodoff, 1979). This paper addresses the question of what occurs at the intersection of these two phenomena; that is, how do expectations about the threat associated with a stimulus influence the deployment of attention to that stimulus?

Expectation based processes have been employed to account for a wide variety of behavioral and neural phenomena, most prominently in the field of reward based learning (Schultz et al., 1997), but also in a range of other areas including non-reward learning (den Ouden et al., 2009), perception (Egner et al., 2010), social inference (Behrens et al., 2008) and overt attention (Itti and Baldi, 2009). Overall, these studies suggest that the brain updates expectations about some aspect of the environment by comparing them with the actual outcome experienced (Friston et al., 2006). The extent to which an observation leads to changes in expectation is proportional to how surprising the observation is (Itti and Baldi, 2009; MacKay, 2003). In other words, expectations are incrementally altered by transient surprise signals which give a measure of the distance between the expectation and a particular experience. Learning can be viewed as a process by which expectations are altered to minimize surprise and thus to more closely resemble experience (Friston et al., 2006). Extrapolating from these observations, we propose that a similar process generates expectations about the probability of threat occurring in the environment. The evolutionary advantage provided by being able to predict the occurrence of threat is clear, however it may also be of clinical interest as deficient control of attention to threat has been described as a causal process in anxiety (Bishop, 2007). We predict that attentional resources should be deployed toward stimuli which are expected to be threatening (regardless of whether they are or not) and toward stimuli which are surprising (regardless of how threatening they are). In addition higher levels of anxiety are predicted to be associated with a sub-optimal use of threat-expectation in the control of attention.

**Fig. 1:**
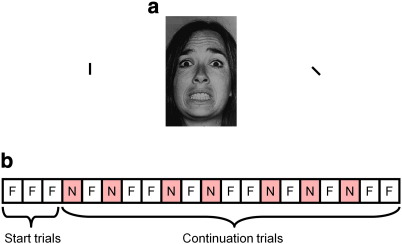
a) Sample trial from the task completed during the study. A centrally presented face which could be fearful or neutral was flanked by two bars and presented for 200 ms. In alternate blocks participants were required to report the gender of the face or whether the flanking bars were aligned. b) Example sequence of trials from a single high-threat block of the task. Each trial displayed either a fearful (F) or neutral (N) face. The first three (start) trials were always of the same expression and in the remaining (continuation) trials the same expression was more common. All blocks were either high-threat as shown or low-threat, in which the neutral faces were more common.

In order to test these predictions we presented participants with a succession of pictures of faces which indicated the presence of threat (a fearful expression) or no threat (a neutral expression). Expectation of threat was experimentally manipulated by varying the relative frequencies of the two expressions (fear or neutral) throughout the course of the study. Variation in blood oxygen level dependent (BOLD) signal in the visual cortex (Vuilleumier, 2005) was used as an imaging measure of the trial-by-trial changes in visual attention to the stimuli. This was supplemented by a reaction time measure of attention (Posner and Petersen, 1990) which was made possible by using two different task instructions; on some trials participants had to respond to a feature of the face (gender) whereas on others they were required to respond to small bars which flanked the face (Fig. 1a). Comparing reaction times on these two types of trial gives a measure of the degree to which attention is directed toward the face as increased attention would be expected to reduce reaction time on the gender task while increasing reaction times on the bars task. An optimal Bayesian learner (Behrens et al., 2007) was presented with the same information as the participants regarding the valence of the faces and used this to generate estimates of threat-expectation and the associated surprise in response to the stimuli. The ability of these model derived estimates to explain the trial-by-trial allocation of attentional resources to the stimuli was then assessed. Having identified the presence of these model derived signals in both the reaction time and visual cortical data, the broader network of cortical regions presumed to be involved in generating the signals was explored. Lastly, the relationship between the use of expectancy to control attention and individual differences in trait anxiety was examined.

## Methods

### Participants

29 participants (18 female) who had been screened to exclude current or previous axis I psychiatric disorder or alcohol/substance misuse using the Structured Clinical Interview for the DSM-IV (Spitzer et al., 2002) were recruited to the study. Participants were also excluded if they were taking any psychoactive medication, had any significant neurological condition or were familiar with any of the tasks or stimuli used in the study. The participants were right handed and provided written informed consent to participate in the study which had been approved by a local ethics board.

### Procedure

Participants completed a single scanning session during which both behavioral and neuroimaging data were collected. Before the scan participants completed the trait subscale of the Spielberger State-Trait Anxiety Inventory (trait-STAI; Spielberger et al., 1983) as well as one of two possible versions of a computer task, designed to encourage attentional deployment toward or away from negative stimuli. The between-subject influence of these pre-scan tasks has been dealt with previously (Browning et al., 2010) and is not considered in the current paper which examines the within-subject variation of attentional deployment.

### Task

During scanning, participants completed a modified version of the task described by Pessoa et al. (2005) (Fig. 1). The task consisted of 8 blocks of 20 trials. On each trial a picture of either a fearful or neutral face (Ekman and Friesen, 1976; Lundqvist et al., 1998; Tottenham et al., 2009) was presented, flanked by two bars, for 200 ms. Stimuli were selected at random, without replacement, from a pool of 88 fear and 88 neutral faces (50% female). Stimuli were presented in a jittered fashion using an exponential function (minimum ISI 6 s, maximum 12 s). During a given block participants were required to report via button box either on the gender of the face or whether the bars were aligned (each outcome occurred on 50% of trials). Varying the task instruction in this manner provides a behavioral measure of attentional deployment while controlling for overall change in response time. Specifically, increased attention directed toward the faces would be expected to reduce reaction time on the gender task and increase reaction time on the bars task. The blocks also varied in regard to the frequency of fearful vs. neutral faces presented (high vs. low-threat blocks; see Fig. 1). The sequence of trials within a block was such that the first three trials were always of the same valence (fear in a high-threat block and neutral in a low-threat block), following this the majority (10 out of 17) of subsequent trials also displayed the dominant expression (Bishop et al., 2004). The specific sequence of trials within a block was selected from 8 predetermined pseudorandom schedules which conformed to the above rules. These schedules were randomly selected within the constraints that for a given participant there were two blocks each of the four possible block types (i.e. high-threat gender-task, low-threat gender-task, high-threat bars-task, low-threat bars-task). The initial task required of participants was to identify the gender of the face and this alternated with the orientation of bars task in successive blocks.

### Computational model

The computational model used generates, within a Bayesian framework, an optimal assessment of the probability that some binary event will occur in the following trial, given the preceding history of trials in which the event was either present or absent (Behrens et al., 2007). In keeping with standard models of associative learning, such as that described by Rescorla and Wagner (Rescorla and Wagner, 1972), the model updates its belief that the event will occur in response to the information presented in each trial. A key difference between the current model and that described by standard models is how it addresses the question of how much this belief should be updated on each trial. Standard models tend to deal with this issue by adding a free parameter, the “learning rate”, which must be set by the experimenter. In contrast, the model used in the current study alters this value in response to the variability of the information it has received; if all trials have tended to present the same information it will update its prediction by only a small amount, whereas if the trials have presented conflicting information it will update its prediction by a greater amount. An advantage of this approach is that it requires no post-hoc fitting of model parameters to participants' data. In summary, for the purpose of the current study, the computational model may be considered as a variant of the Rescorla–Wagner learning rule with the learning rate parameter being inferred from the data rather than being set by the experimenter.

The model was provided with information about whether a threatening (fearful) or neutral face was presented on each trial in a block of the task and used this information to update its expectation that a threatening face would be presented on the following trial. The surprise associated with a trial was defined as the negative logarithm of the conditional probability of the observation, given the expectation (sometimes called the self-information; MacKay, 2003).^1^ Model derived threat-expectation and surprise regressors were created for each individual participant in the study, using the particular sequence of trials displayed to that participant. Importantly, the magnitudes of the expectation and surprise regressors were not correlated (highest correlation of individual regressors; *r* = 0.007, *p* = 0.93).

### Image acquisition

A BOLD contrast signal was acquired across the whole brain using echo planar imaging on a 3 T Siemens TIM Trio System (Siemens, Erlangen, Germany). A total of 45 slices were acquired using a voxel resolution of 3 × 3 × 3 mm^3^, repetition time = 3 s, echo time = 30 ms, flip angle = 87°. The slice angle was set to 30°. During the task 405 volumes were collected. T1-weighted structural images of the whole brain were acquired for subject alignment using a magnetization prepared rapid acquisition gradient echo sequence with the following parameters: voxel resolution 1 × 1 × 1 mm^3^, echo time = 4.7 ms, repetition time = 2040 ms, 192 slices collected.

### Data analysis

#### Behavioral data

In order to test the ability of the model derived regressors to predict a behavioral measure of attention, separate general linear models were fitted to each participant's reaction time data. Before performing these analyses the positive skew of the reaction time distributions was reduced by removing extreme responses (less than 200 or greater than 1400 ms; mean data loss 7%) and log transforming the data. As described above, increased attention toward the facial stimuli should be indexed by increased reaction time on the bars task and decreased on the gender task. Therefore the critical explanatory variables in these analyses coded for the interaction between the model derived regressors and the task demands (threat-expectancy × task and surprise × task). Importantly, the analyses controlled for all other relevant aspects of the task by including additional regressors which coded for: a) the emotion of the face presented (fear or neutral), b) the task being performed (gender identification or bars aligned), c) the model derived expectancy and d) surprise signals, e) whether the participant made an error on that trial and f) the interaction between the emotion of the face and the task being performed. These multiple regression analyses, performed individually for each participant, resulted in estimates of the regression coefficients for each explanatory variable, for each participant. A second level analysis then tested whether the two critical regression coefficients significantly differed from zero across the group of participants, and whether this difference was in the predicted direction (using two-tailed, one sample t-tests). Analysis was performed using the statistical toolbox of Matlab (R2009a; The Mathworks Inc., Natick, MA).

#### Imaging analysis

Functional magnetic resonance imaging analysis was carried out using FEAT (FMRI Expert Analysis Tool) Version 5.91 (www.fmrib.ox.ac.uk/fsl). Image pre-processing employed the default options for FEAT: motion correction was applied using rigid body registration to the central volume; Gaussian spatial smoothing was applied with a full width half maximum of 5 mm; brain matter was segmented from non-brain using a mesh deformation approach; high pass temporal filtering was applied with a cut off of 100 s.

The critical regressors in the first level model for the imaging analysis were the demeaned model derived expectancy and surprise signals. These regressors were timelocked to the onset of the appropriate face stimuli; the events coded by the expectancy regressor had a duration of 4 s, the events coded by the surprise regressor had the same duration as the faces presented (0.2 s). Different durations for the expectancy and surprise regressors were chosen to reflect the roles of the two forms of information within the model. Specifically, in order for learning to proceed, the model must carry forward the estimate of threat-expectation from one trial to the next, in contrast the surprise signal is involved in the transient updating of threat-expectation. The effects of other aspects of the task were controlled for by including regressors (and their temporal derivates) coding for the presentation of each of the four basic trial types (fear gender-task, fear bars-task, neutral gender-task, neutral bars-task) as well as for trials where an error was made. The addition of these extra regressors ensures that activations attributed to the model derived regressors cannot be accounted for by participants' responses to stimuli presentation or to the emotion presented on the faces.

A random effects analysis was employed at the group level to assess for regions in which the BOLD signal covaried with the model derived threat-expectation and surprise regressors across all participants. Trait anxiety, as measured by trait-STAI scores, was added as a covariate in this analysis to assess whether varying levels of trait anxiety were associated with varying use of expectancy and surprise to control attention. Note that, in contrast to the behavioral analysis, the effect of attention to the faces was not expected to interact with task instructions; in other words, increased attention to the faces was expected to cause increased visual cortical activity regardless of whether the task required participants to respond to the gender of the faces or the orientation of the bars. Cluster based multiple comparison correction with an initial Z-threshold of 2.3 (equivalent to a *p* of 0.01) and correction over the whole brain using *p* < 0.05 was used. For the identified clusters, MNI co-ordinates provide the location of the peak voxel within the cluster, Z-max is the z score for this voxel and the *p* value is corrected at the cluster level.

## Results

### Behavioral data

**Fig. 2:**
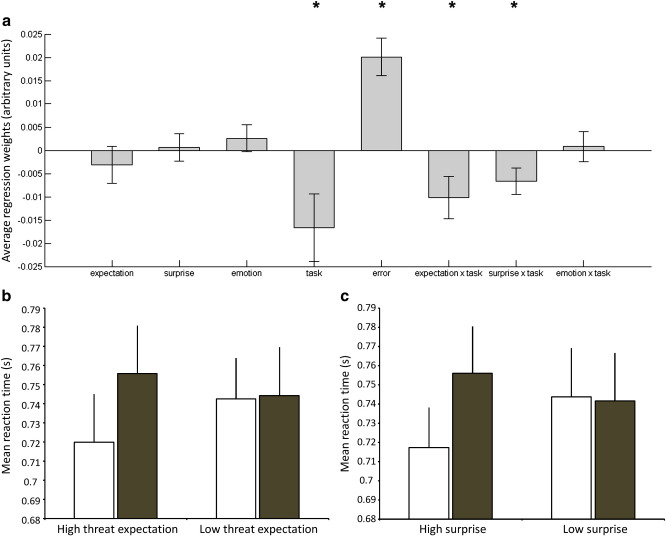
Results of behavioral analysis. a) Average regression weights from the analysis of reaction times. Regression analyses were run on each individual's reaction time data using the explanatory variables listed along the x-axis. The critical explanatory variables (expectation × task, surprise × task) tested whether participants displayed the predicted modulation of reaction time associated with threat-expectancy and surprise (see methods section). * = p < 0.05. The average reaction time of trials in which b) threat-expectation or c) surprise was high (in the top quartile of the range within a given participant) or low (in the bottom quartile) were calculated separately for the gender task (white columns) or the bars task (gray columns). As can be seen, higher values of both of these regressors were associated with increased reaction time on the bars task and decreased reaction time on the faces task. Error bars represent standard error of the mean.

Both the model derived threat-expectancy and surprise signals had the predicted effect on the deployment of attention as assessed using reaction times. Specifically, reaction times were increased in the bars relative to the gender task on trials in which the model expected the face to be threatening [expectation × task; *t*(28) = 2.24, *p* = 0.03] and on trials in which the face presented was surprising [surprise × task; *t*(28) = 2.34, *p* = 0.03]. In addition (Fig. 2), participants were also generally slower on trials in which they made an error [*t*(28) = 5.0, *p* < 0.001] and when completing the bars rather than the faces task [*t*(28) = 2.3, *p* = 0.03]. There was no correlation between levels of trait anxiety and either the threat-expectation × task [Pearson's *r*(29) = − 0.14, *p* = 0.5] or surprise × task regressors [Pearson's *r*(29) = 0.02, *p* = 0.9]. Average reaction time did not significantly alter across successive blocks for the faces task [*F*(3,84) = 1.1, *p* = 0.35] or bars task [*F*(3,84) = 1.6, *p* = 0.19].

### Imaging data

#### Threat expectation

**Fig. 3:**
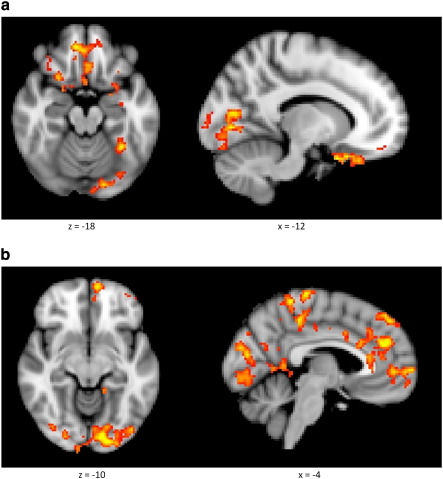
Results of whole brain analyses showing activity associated with the threat-expectation and surprise regressors. a) Threat-expectation was found to covary with activity in the left ventral visual stream and with attentional control regions including the OFC and left amygdala. b) Surprise was associated with activation of the visual cortex and a network of frontal regions including the ACC, dlPFC and ventral striatum (not shown). Average activations maps across all participants have been rendered over the standard MNI brain.

Consistent with the behavioral analysis, a neuroimaging measure of the deployment of attention, activity in the visual cortex, was increased when threat-expectation was high (Fig. 3a). A cluster in which activity covaried with the threat-expectancy regressor was identified extending throughout the left ventral visual processing stream from occipital pole to temporal–fusiform cortex [*x y z* = − 8 −80 −2, *Z*-max = 3.93, *p*-corrected < 0.001]. The whole brain analysis also identified a number of attentional control regions in which activity also covaried with the threat-expectation signal. These included bilateral orbitofrontal cortex [OFC; *x y z* = 12 46 −16, *Z*-max = 3.67, *p*-corrected = 0.002] and a left sided fronto-temporal cluster [*x y z* = − 30 24 −30, *Z*-max = 3.85, *p*-corrected = 0.001] which extended into the left amygdala. Additionally a posterior parietal cluster was identified, on the left hand side [*x y z* = − 36 −50 28, *Z*-max = 3.91, *p*-corrected = 0.004]. There was no effect of the task completed by participants (i.e. gender vs. bars) on the relationship between activity and threat-expectation in any of these clusters.

#### Surprise

An extensive network of regions was found to covary with the surprise regressor (Fig. 3b). This included large volumes of both the left and right visual areas including both occipital–fusiform cortices [*x y z* = − 12 −88 −12, *Z*-max = 3.71, *p*-corrected < 0.001] extending forward into the left thalamus, an extensive frontal midline cluster incorporating both dorsal and rostral ACC [*x y z* = − 2 44 28, *Z*-max = 3.53, *p*-corrected < 0.001], left sided dlPFC [*x y z* = − 14 66 2, *Z*-max = 4.02, *p*-corrected = 0.006] and precentral gyrus [*x y z* = − 2 −28 72, *Z*-max = 3.71, *p*-corrected = 0.02]. These activations were not significantly modified by the task completed by participants.

#### Association between visual cortical activations and face processing

As the facial stimuli are significantly more complex than the bars, the visual cortical activations reported above are likely to reflect increased attention to the faces. This assumption was further tested using a conjunction analysis which compared the results reported above with the effects of task instruction. A simple comparison of the trials from the gender-task blocks (which require attention to be directed to the face) with those from the bars-task blocks (which require attention to be directed away from the face) across the whole brain revealed a single cluster of activation in the occipital pole [*x y z* = − 18 −104 0, *Z*-max = 3.38, *p*-corrected = 0.02]. This cluster overlapped with the visual cortical activations of both the threat-expectation and surprise regressors (in fact the visual cortex was the only region in which the expectation and surprise activations overlapped). Thus increased attention to the faces induced by task instruction produced increased activity in the same regions of visual cortex as that associated with both the threat-expectancy and surprise regressors.

#### Influence of trait anxiety

**Fig. 4:**
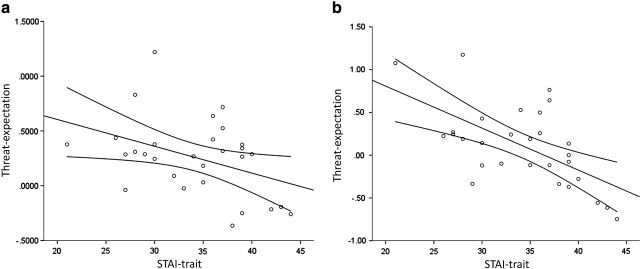
Scatterplots demonstrating the negative correlation between trait anxiety and utilization of the threat-expectation information in a) the region of the visual cortex identified in the conjunction analysis as being responsive to both threat-expectation and surprise and b) a structural mask of the left amygdala. Estimated regression lines with 95% confidence intervals have been added to the plots.

A number of regions displayed a negative correlation between trait anxiety and utilization of the threat-expectancy signal. These included the right posterior parietal and lateral occipital cortex [*x y z* = 32 −68 52, *Z*-max = 3.78, *p*-corrected < 0.01], right side dlPFC [*x y z* = 50 8 18, *Z*-max = 3.66, *p*-corrected < 0.01], right [*x y z* = 32 16 −10, *Z*-max = 3.84, *p*-corrected < 0.01] and left vlPFC [*x y z* = − 34 24 −6, *Z*-max = 3.78, *p*-corrected < 0.01], medial PFC including the dorsal and rostral ACC [*x y z* = 4 42 28, *Z*-max = 4.15, *p*-corrected < 0.01] and the posterior cingulate/precuneus [*x y z* = 8 −32 28, *Z*-max = 3.58, *p*-corrected = 0.02]. These regions overlapped with those identified as responding to threat-expectancy generally in the right vlPFC (32 26 −10), left temporal lobe abutting the amygdala (− 32 −8 −18) and precuneus (10 −56 16). A similar negative correlation was found when analysis was limited to the visual cortical cluster (see Fig. 4), identified in the conjunction analysis reported above [Pearson's *r*(29) = − 0.4, *p* = 0.03]. No regions displayed a positive correlation with trait anxiety.

Consistent with the decreased use of threat-expectancy to guide attention in high levels of anxiety, trait anxiety was found to correlate positively with surprise in a single cluster in the left superior temporal gyrus cortex [*x y z* = − 52 −44 6, *Z*-max = 3.74, *p*-corrected = 0.03] with no regions displaying a negative correlation. However, this region was not identified in the initial analysis as responding to either expectancy or surprise.

#### Influence of prescan manipulation

We did not expect the prescan manipulation of attention to negative stimuli (Browning et al., 2010) to interact with the effects of either threat-expectation or surprise. Consistent with this, between subject t-tests revealed no significant difference between the two forms of prescan manipulation for the behavioral measures of threat-expectation or surprise [*t*(27) < 1.8 *p* > 0.09] and the neuroimaging analysis also revealed no significant effects.

## Discussion

The current study demonstrates that a simple expectancy based model is able to account for a significant amount of variance in both the behavioral and neural signatures of attention to emotional faces. Specifically, markers of participants' attention to the faces increased when the computational model expected the faces to be threatening or when it found the emotional content of the faces surprising. The predictive validity demonstrated by the computational model in the current study suggests that similar expectancy based calculations may be instantiated by neural systems in order to influence the deployment of attention to threat. The degree to which individuals employ threat expectancy to control attention may vary as a function of anxiety as increased levels of self-reported trait anxiety was associated with a decreased correlation between the model derived threat-expectancy signal and neural activity in a number of attentional control and visual cortical regions. However, this relationship was not observed in the behavioral data. Lastly, the current results are unlikely to be accounted for by task or stimulus related confounding factors such as the valence of the presented stimuli as both the behavioral and neuroimaging analyses performed were careful to control for such factors.

As well as demonstrating the predicted influence of threat-expectation and surprise in the visual cortex the current study was able to assess other regions of the brain which may be involved in generating and tracking such expectancies. The threat expectation signal itself was found in bilateral OFC and the amygdala on the left, both of which have previously been identified as being involved in the control of attention to emotional stimuli (Vuilleumier, 2005). Interestingly, it has been suggested that both the OFC (O'Doherty et al., 2001; Rolls, 2004; Rudebeck et al., 2008) and the amygdala (Adolphs, 2010; Murray, 2007) code for the value of stimuli in reward guided paradigms. Value is defined as the reward-expectation associated with a stimulus and, although the current study does not assess this, threat-expectation fulfills a similar role in the computational model as value does in reward learning. Thus the OFC and amygdala may perform a similar role in generating and tracking this different form of expectancy. Such a conclusion would be consistent with the observations that both these areas respond to salient negative as well as positive events (Adolphs, 2010; Kringelbach and Rolls, 2004). The surprise signal was associated with extensive activation, particularly in the ACC. This finding is consistent with recent electrophysiological evidence indicating that dorsal ACC neurons respond to the surprise associated with a stimulus in a probabilistic choice task (Hayden et al., 2011). However, perhaps the most striking aspect of the neuroimaging analysis is that the only brain region in which the expectation and surprise signals overlap is the visual cortex. One possible interpretation of this is that the two signals are supported by distinct neural systems, with the surprise signal activating the phasic alertness network (Posner, 2008; Yanaka et al., 2010) and the expectation signal the stimuli value network.

As described above, a negative correlation was found between individual self reports of trait anxiety and the threat-expectation signal in a range of attentional control and visual cortical structures. There was also evidence of a positive correlation between trait anxiety and the surprise signal, although this was in an area which had not been identified in the main analysis. Anxious individuals, therefore, appeared less able to utilize the statistical regularities of their environment to predict the occurrence of threatening stimuli. Interestingly, previous work has demonstrated that exposure to unpredictable environments increases anxiety (Grillon, 2002; Mineka and Oehlberg, 2008). This raises the possibility that the apparent deficit in threat prediction may have caused the increased symptoms of anxiety—as an inability to predict when threat will occur will, in effect, expose an individual to an unpredictable environment. However, an important caveat to this interpretation which should be acknowledged is that there was no evidence that anxiety interacted with threat-expectancy when assessed using the behavioral measures of attention, which would have strengthened the interpretation of the neuroimaging data.

The regressors used in the current paper were generated by a computational model which had been presented with the same information on face valence as the participants. It is possible to create similar regressors without the input of a computational model, by using knowledge of the task structure; specifically, the model derived threat-expectancy regressor is similar to a task derived regressor which codes for whether a trial occurs in a high or low threat block and the surprise regressor is similar to the absolute difference between this and a regressor coding for the face valence on each trial. Analyzing the data using such task derived regressors produces similar results to those reported above. Crucially, however, the participants had no prior knowledge about task structure. In this context, the advantage of using the computational model is that it demonstrates, formally, how participants may have generated the estimates of threat-expectancy. An example of the insights provided by this approach is the surprise signal found in the data; surprise is not an obvious parameter of the experimental task, its relevance being suggested by the expectancy based model. The finding that activity in an extensive neural circuitry covaried with surprise suggests that the brain may instantiate a similar computational process to that described by the model.

Finally some limitations to the current study should be acknowledged. Firstly, our task did not allow a conclusive examination of the degree to which activity in the visual cortex reflected processing of the facial stimuli. While the conjunction analysis was able to demonstrate that manipulation of attention to the faces produced by task instructions altered activity in the same region of cortex as that influenced by both threat-expectancy and surprise, alternative task designs may have facilitated a finer grained analysis of face processing. For example future studies may benefit from pairing face stimuli with pictures of houses, which are thought to invoke activity in distinct regions (Egner et al., 2010), and thus may allow firmer conclusions about the specificity of the observed activations. Secondly, all participants were initially required to complete the faces task, with subsequent blocks alternating between this and the bars task. Ideally, the order of these tasks should have been counterbalanced across participants. This aspect of the study design does not impact on the imaging analysis, as this measure of attentional deployment was invariant across the two task instructions, however if there was a consistent effect of block position on reaction time, it may have influenced the reaction time analysis. Reassuringly, analysis of reaction times across the blocks revealed no evidence of an effect of block position for either of the task instructions.

## Conclusion

In the current study, a computational model of threat-expectancy was used to predict the behavior and neural activity of non-clinical participants who were presented with emotional stimuli. Attention to the faces was significantly predicted both by model derived estimates of threat-expectation and by the surprise elicited when these expectations were violated. Individuals with higher levels of trait anxiety were less able to modify neuroimaging measures of attention in response to threat-expectation. These results suggest that expectancy based processes may plausibly be involved in the control of attention to threatening information and that these processes are perturbed in anxiety.

## Footnotes

1 In reinforcement learning paradigms, surprise is sometimes defined as the magnitude of the prediction error associated with a stimulus (e.g. den Ouden et al., 2009). In the current study prediction error magnitude (i.e. the absolute difference between expectation and outcome) results in a virtually identical regressor to the self-information regressor used in the analysis and does not alter the results.
